# Investigation of the Mechanical Properties and Microstructure of the Co40NiCrMo Alloy Used for STACERs and Prepared by the CSPB Process and the Winding and Stabilization Method

**DOI:** 10.3390/ma16082970

**Published:** 2023-04-08

**Authors:** Ruilong Lu, Jingtao Han, Jiawei Liu, Zhanhua Li, Congfa Zhang, Cheng Liu, Xiaoyan Ma

**Affiliations:** 1 School of Materials Science and Engineering, University of Science and Technology Beijing, Beijing 100083, China; 2 Guangzhou Sino Precision Steel Tube Industry Research Institute Co., Ltd., Guangzhou 511300, China; 3 School of Mechanical Engineering, Shijiazhuang Tiedao University, Shijiazhuang 050043, China; 4 Institute of Spacecraft System Engineering, China Academy of Space Technology, Beijing 100094, China

**Keywords:** STACER, Co40NiCrMo alloy, mechanical property, residual stress, microstructure

## Abstract

The Co40NiCrMo alloy, used for STACERs fabricated by the CSPB (compositing stretch and press bending) process (cold forming) and the winding and stabilization (winding and heat treatment) method, was investigated with regard to its tensile property, residual stress, and microstructure. The Co40NiCrMo STACER prepared by the winding and stabilization method was strengthened with lower ductility (tensile strength/elongation: 1562 MPa/5%) compared to that prepared by CSPB (tensile strength/elongation: 1469 MPa/20.4%). The residual stress of the STACER prepared by winding and stabilization (τ_xy_ = −137 MPa) showed consistency with that obtained through CSPB (τ_xy_ = −131 MPa). Combined with the driving force and pointing accuracy performances, the optimum heat treatment parameters for the winding and stabilization method were determined as 520 °C + 4 h. The HABs in the winding and stabilization STACER (98.3%, of which 69.1% were Σ3 boundaries) were much higher than those in the CSPB STACER (34.6%, of which 19.2% were Σ3 boundaries), while deformation twins and h.c.p ε-platelet networks were present in the CSPB STACER, and many more annealing twins appeared in the winding and stabilization STACER. It was concluded that the strengthening mechanism in the CSPB STACER is the combined action of deformation twins and h.c.p ε-platelet networks, while for the winding and stabilization STACER, annealing twins play the dominant role.

## 1. Introduction

STACERs (spiral tubes and actuators for controlled extension and retraction), which are typical one-dimensional linear space deployment mechanisms with advantages, such as high exhibition ratio, high deploying accuracy, simple structure, small size, lightweight, self-driving and self-stiffening properties, strong circumferential thermal symmetry, etc., are becoming increasingly popular among various spacecrafts [1,2,3,4].

**Figure 1:**
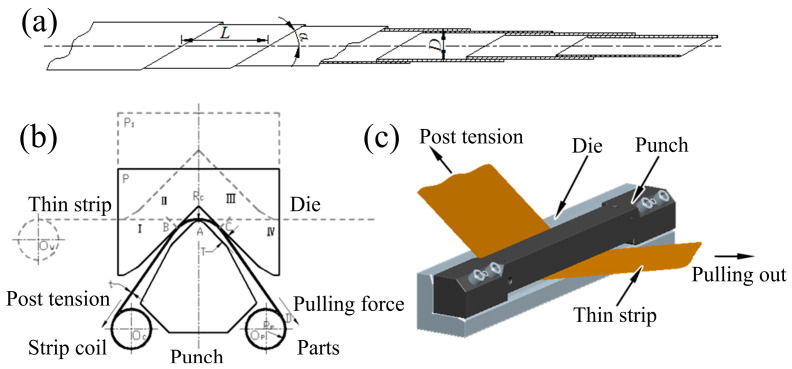
Schematic plot of STACER (**a**), the principle of the CSPB process (**b**), and its model diagram (**c**).

A STACER is spirally fabricated from a thin flat metal strip, rolled with a constant diameter (D), helical pitch (L), and fixed helix angle (α), as shown in Figure 1a. STACERs range in size from 1–10 m in length to 4–55 mm in diameter at the tip, and can provide extensive force of almost nothing to >200 N [5]. A STACER integrates the power generator, transmission pair, and actuator into the component itself compared with other split space deployable mechanisms. In the process of deployment in orbit, it can provide a nearly constant driving force due to its internal elastic potential energy without any external driving force. The stowed STACER is a coil of thin strips; during the process of deployment, the latter layer strip tightly covers the former layer with a certain overlap ratio, and the strip under the geometrical constraints coupled with the interlayer contact friction eventually forms the STACER, which is like a thin wall tube with enough stiffness, size accuracy, and service stability.

The current most well-known method for forming STACERs is the CSPB (compositing stretch and press bending) method proposed by Li [6,7]; other methods are not as clear in detail, such as that proposed by Wu [8]. In the CSPB process, the forming tools consist of a pair of a punch and die; a thin strip is pulled out of the clearance of the forming tools, and STACERs with various diameters are obtained by adjusting the size of the mold clearance. One of the most notable features of CSPB is that forward bending and reverse bending are introduced to guarantee size accuracy and deploy precision and stability. Key forming parameters such as post-tension, die gap, punch radius, and strip thickness have been well studied, as well as the mechanical properties of the STACER as an aerospace component, as elaborated in the literature [6,9,10]. Yu et al. [11] proposed a method for simulating the STACER’s deployment deformation, in which a variational method was adopted. Based on the deformation characteristics of the steel strip, a proper and possible shape function space was established to calculate the principle stress and deformation energy due to the variation pattern of the extension length of the STACER and, finally, the deploying force was obtained by using the principle of minimum potential energy and the principle of virtual work. Li et al. [12] conducted the analysis and verification of the deployed stiffness of a STACER and found that the natural frequency first mode and specific stiffness changes with different tip radii, tip helix angles, strip widths, and strip thickness of the STACER. Kong et al. [13] analyzed the influence of top rod radius on the gathering process and the effects of surface friction coefficient and acceleration on stability during the gathering process of a STACER.

The classical materials used for manufacturing STACERs are Co40NiCrMo, beryllium copper, stainless-steel strips, and so on. Co40NiCrMo is the most widely used alloy for STACERs; some researchers [14] investigated the strengthening mechanism of the Co40NiCrMo alloy subjected to solid solution and cold drawing treatment by using transmission electron microscopy (TEM), and it was found that the slim deformation twins and their network structure after cold drawing significantly increased the strength of the alloy, as did the formation of a Suzuki atmosphere due to the segregation of Cr, Mo, and C atoms after the aging process. In the emerging technology, more studies need to be conducted on the surface improvement of STACER, such as the study of pulsed laser [15] and 2D structure material [16] on the surface of STACER, which are considered to be prospective research projects for improvement of the electric, thermal, mechanical, and radiation resistant properties of STACER on orbit.

To explore the residual stress distribution state, the X-ray diffraction technique for residual stress measurement can be used, which is usually associated with sin^2^ Ψ, a method based on the interception of the diffraction cone and line detectors. To overcome the loss of information, cos α, an alternative method using a single exposure to collect the entire diffraction cone via a 2D detector, was employed [17]. The present paper compared both the sin^2^ Ψ and cos α methods in the residual measurement of STACER and determined the values for STACERs with good service behaviors.

The STACER research mentioned above mainly focuses on the manufacturing process and the mechanical or service performance; few studies have been conducted on the STACER microstructure. On that note, the Co40NiCrMo STACER, as an elastic boom based on Co40NiCrMo strips, should receive more attention with regard to its residual stress distribution state and microstructure characteristics [18] before and after the formation of the STACER. In this paper, in addition to the CSPB forming process, a new method of manufacturing STACERs is proposed—winding and stabilization—which saves more time, controls the forming process more easily, and is easier to automate. Further, the STACER of this process has sound service behaviors compared to the CSPB STACER. The tensile properties of Co40NiCrMo strips for STACERs were analyzed followed by fracture morphology observation through scanning electron microscopy (SEM). Then the residual stress was tested through the X-ray diffraction residual stress measurement method, and the microstructure of STACER was investigated by electron back-scattered diffraction (EBSD) and transmission electron microscopy (TEM).

In brief, unlike the above previous studies on the service performance and optimization of STACER products, this paper attempts to explore the changes in mechanical properties, residual stresses, and microstructure of the Co40NiCrMo alloy after deformation at the micro level, aiming to identify the strengthening mechanism of the Co40NiCrMo alloy used for STACERs prepared by different forming methods.

## 2. Materials and Methods

### 2.1. Material

The raw material used in this study is a Co40NiCrMo (Elgiloy or 3J21 alloy) thin alloy strip manufactured by cold rolling process (reduction of about 70%); the strip width is 127 mm and the thickness is 0.15 mm. The chemical composition (wt.%) is presented in Table 1.

### 2.2. Forming Process

**Figure 2:**
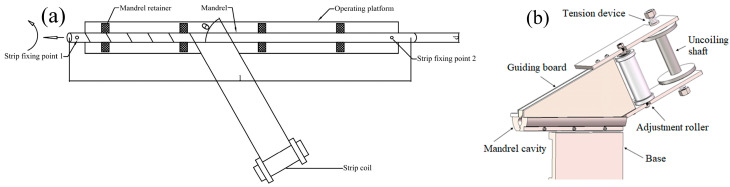
The schematic diagram of the winding procedure (**a**) and its core model structure (**b**).

**Figure 3:**
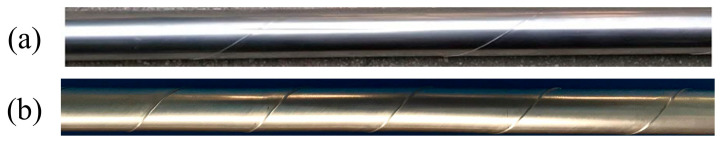
Physical pictures of STACERs obtained by (**a**) CSPB method and (**b**) winding and stabilization method.

Two manufacturing processes were utilized to obtain STACER, and the forming principles are described as follows: (1) CSPB process, which follows the forming principle shown in Figure 1b,c. The thin alloy strip is first made through the clearance of the punch and die, then the punch and die are tightened with the given clearance, the post-tension is imposed on the strip at the inlet side, and gradually the thin strip is pulled out at an angle to the longitudinal direction of the forming tools by the pulling force at the outlet side. Then, the deformed strip is collected at the receiving shaft, and is eventually released from the shaft and forms the STACER. Since the CSPB process belongs to the cold forming method, we also call CSPB STACER cold-formed STACER. (2) The winding and stabilization process, which has fewer procedures, a higher finishing rate, and easier control than the CSPB method, is divided into two steps: the winding step and the stabilization step. The winding step is schematically shown in Figure 2. The two ends of the mandrel are tightened to the three jaw chucks of the equipment, and one end of the thin strip is fixed at strip fixing point 1 at a certain given angle to the longitudinal direction of the mandrel axis. With the rotation and horizontal movement of the mandrel, as the arrow indicates in Figure 2a, the strip is gradually wound on the mandrel shaft and then fixed tightly at strip fixing point 2, forming the pre-formed part. The final forming part is obtained after the shaping procedure of stabilizing heat treatment for the pre-formed part. The physical images of STACERs obtained by two methods are shown in Figure 3.

### 2.3. Tensile Experiment and Fracture Morphology Observation

**Figure 4:**
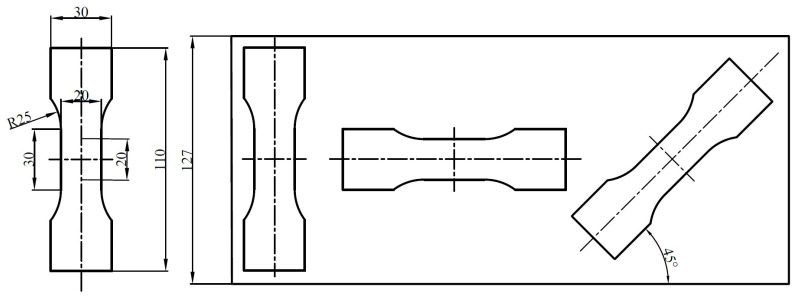
The schematic plot of tensile specimen and cutting directions of strips (Unit: mm).

The tensile specimens are raw strips and CSPB STACER, while the winding and stabilization process consists of stabilizing heat treatment procedure (at 480 °C, 520 °C, and 560 °C holding for 2 h, 3 h, and 4 h respectively). The specimens of the winding and stabilization STACER are obtained according to the parameters of heat treatment. The dimensions and cutting directions (longitudinal direction, transverse direction, and the 45° direction with the strip rolling direction) of all tensile specimens are shown in Figure 4. The tensile experiments were conducted on a universal material testing machine, CMT 4204 (UTC 2017-042). The fracture morphology observation on the fresh fracture surface was conducted with a ZEISS Gemini SEM 500 instrument.

### 2.4. Residual Stress Measurement

In this paper, two methods are used for the measurement of the residual stress of STACER: sin^2^ Ψ and cos α. (1) Proto iXRD apparatus was used to collect the diffraction peaks for the stress calculation via the sin^2^ Ψ method. The Proto apparatus is limited to seven different incident angles between ±13°; for each incident angle, 30 exposures of 0.25 s were used, so measuring time was 52.5 s. All the diffraction peaks were fitted by using the Gaussian peak fitting method to obtain the value of residual stress. (2) The measurement device used for the cosα method is Pulstec μ-X360n X-ray residual stress analyzer with Cr-tube (X-ray wavelength λ = 2.291 Å, operating voltage is 30KV, operating current is 2mA), which is a compact portable system with a 2D area detector based on the principle that the strains are first determined by the acquired Debye–Scherrer ring and then the residual stress is calculated by the cosine α method [19,20,21]. The Kα doublet from {220} plane’s family was used due to its high Bragg’s angle (2θ = 128.902°) providing better accuracy of measurement; the surface of the STACER was cleaned by ethanol without any other treatment.

### 2.5. EBSD and TEM Experiments

The EBSD measurement was used to study the microstructure and orientation evolution of the STACERs. The apparatus used was an Oxford Instruments Nordlys Max 3. The specimen preparation was as follows: the strips of STACER were cut into slices (5 mm × 5 mm), then mechanically polished after griding through 2000 grit sandpaper, and finally electrolytically polished with 5% alcohol perchloric acid. The TEM method was used to study the evolution of microstructures and the second phase particles or twins on the matrix. The specimen preparation was as follows: the 3 mm diameter disks with a thickness of 0.15 mm were obtained from the strips by using a spark-erosion cutter, pre-thinned to 30 μm thickness. The thinned region was obtained through the Gatan 691 ion-beam thinning device, and the Cu self-supporting grid was used before the testing on the TECNAI F-200 200kV field-emission transmission electron microscope (TEM).

## 3. Results and Discussion

### 3.1. Tensile Experiment Results and Fracture Morphology Analysis

**Figure 5:**
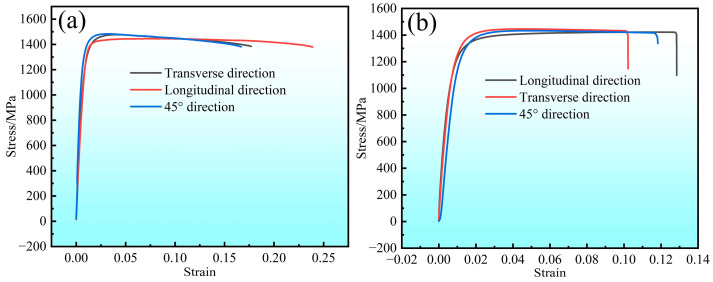
The stress–strain curves of (**a**) raw strips and (**b**) CSPB STACER.

The results of the tensile experiment for raw Co40NiCrMo strips are shown in Figure 5a, and the data are laid out in Table 2. The stress–strain curves show similar patterns with the tensile strength/elongation rate at 1478.09 MPa/17.92%, 1445.83 MPa/24.96% and 1483.22 MPa/18.48% (average value is 1469.04 MPa/20.4%). The longitudinal direction shows a slightly lower value in tensile strength and a higher value in elongation rate than the other two directions. The stress–strain curves of the cold-formed CSPB STACER (see Figure 5b) show almost the same trend as the raw strips. The values of all the tensile strength/elongation rates are 1418 MPa/12.8%, 1415 MPa/11.6%, and 1429 MPa/10.1% (average value is 1421 MPa/11.5%), implying that the CSPB process had little influence on the tensile properties of the cold-rolled raw strips [22].

**Figure 6:**
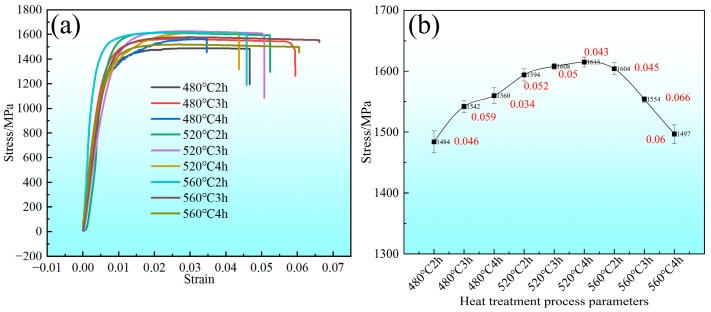
The stress–strain curves (**a**) and stress changes (**b**) according to heat treatment parameters of STACER formed by the winding and stabilization process (elongation: red number).

**Figure 7:**
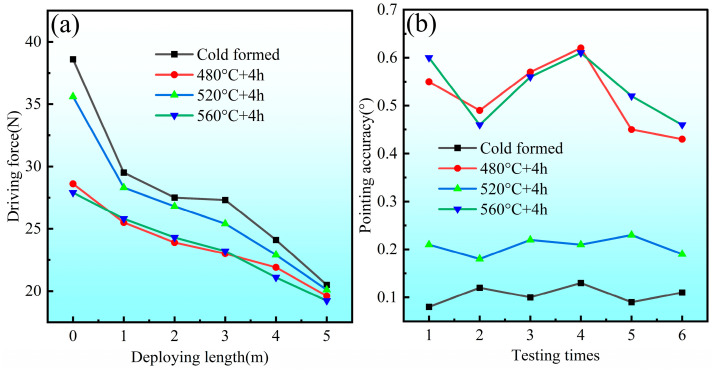
Typical service performances for CSPB STACER (cold formed) and winding and stabilization STACER: (**a**) driving force; (**b**) pointing accuracy.

The stress–strain curves of the STACER formed by the winding and stabilization process are shown in Figure 6a; every individual line stands for a stabilizing heat treatment condition, and the tensile strength ranges from 1484 MPa to 1615 MPa (average: 1562 MPa) with an average elongation rate around 5%. Another view of the tensile property of these specimens is plotted in Figure 6b; it was found that, with the increase in heating temperature and holding time, the tensile strength increases first and then decreases, and the turning point is 520 °C + 4 h, similar to the mechanism illustrated by the previous study [23]. This trend depends on the microstructure changes in the alloy; with the increase in heating temperature and the holding time, the amount of precipitated phases increases, and the more uniform the distribution, the more obvious the strengthening effect. When the heating temperature is higher than 520 °C and the holding time is more than four hours, the precipitated phase dissolves, the strengthening effect is reduced, and the recovery and recrystallization are accelerated. Moreover, the STACERs formed under this condition have consistent service performances, such as pointing accuracy and driving force (see Figure 7, details in Table 3, STACERs obtained under 520 °C + 4 h with the closest service performance to the CSPB STACERs; testing procedures from the literature [6]) with the STACER formed by the CSPB process, which have already been applied in the aerospace industry [24]. As we know, the excellent properties of materials mainly depend on the optimization of the microstructure; the STACER formed by the stabilization parameter at 520 °C + 4 h with good service performance also relates to its special microstructure [25], which is discussed later.

**Figure 8:**
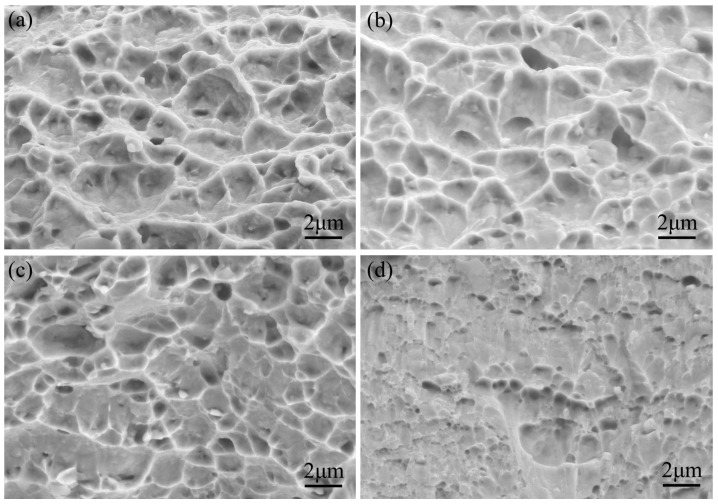
The fracture morphology after the tensile experiment of the Co40NiCrMo strips: (**a**) CSPB STACER; (**b**–**d**) winding and stabilization STACER under 480 °C + 4 h, 520 °C +4 h, and 560 °C +4 h, respectively.

The fractography is laid out in Figure 8; the cold-formed STACER after the tensile test shows equiaxial dimples with craters of varying sizes and depths, since the number of dimples per unit area on the fracture surface depends on the number of nucleation sites and the plasticity of the material. If many nucleation sites were present, void growth would be limited because of the intersecting and linking up of neighboring dimples [26,27]. The SEM images indicated that the fracture mechanism is mainly ductile due to the weakness of the grain boundaries and the aggregation of micropores, which is indicative that the Co40NiCrMo strip has high strength and relatively good ductility [28,29]. The microscopic fractography of the Co40NiCrMo STACER obtained by the winding and stabilization process is shown in Figure 8b–d, which showed that, with the increase in heating temperature, the depth of the dimples became shallower, and the craters became smaller. In this condition, the number of dimples per unit area on the fracture surface depends on the number of nucleation sites, the number of grains, and the plasticity of the material. Many cracks formed at the grain boundaries and intergranular cracking occurred by the growth and coalescence of the micro voids along the grain boundary during the tensile test; the dominant mechanism of ductile fracture for the Co40NiCrMo is the coalescence of the high density of nucleated voids. The change in failure behavior could be dictated by the interaction between the dislocation movements [30]. As shown in Figure 7, after the process parameter of 520 °C + 4 h, the strength decreased and the toughness increased [31]; combined with the tensile experiment results and the service performance of the STACER, we set 520 °C + 4 h as the optimum parameters for stabilizing heat treatment.

### 3.2. Residual Stress Analysis

**Figure 9:**
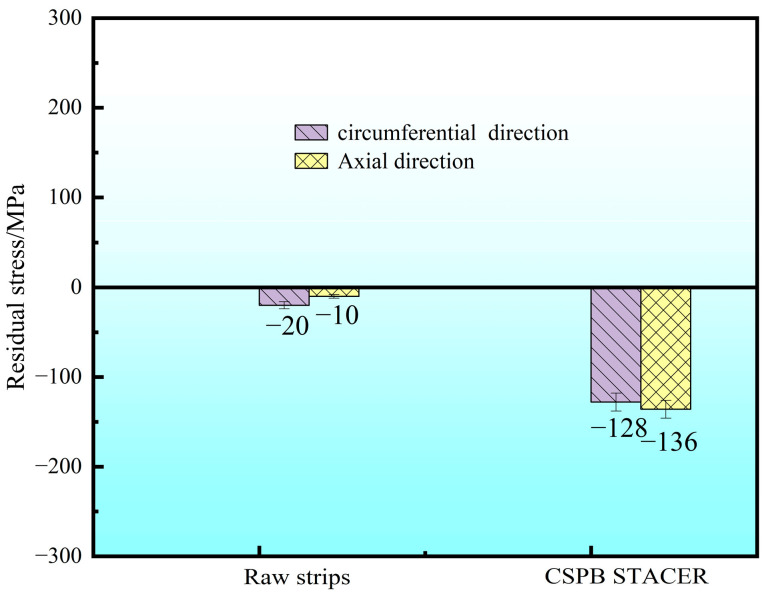
The residual stress results of raw strips and CSPB STACER using sin^2^ Ψ method.

The raw strips and CSPB STACER were tested for residual stress by the sin^2^ Ψ method; as shown in Figure 9, the residual stress values of the raw strips were −10~−20 MPa, while for the cold-formed STACER, the residual stress components were −128 MPa (τ_xy_) for the circumferential direction and −136 MPa (σ_x_) for the axial direction. After the cold forming process, due to the larger angle of the rolling direction of strips to the axial direction of the STACER (see α angle in Figure 1a.), larger residual compressive stress (mainly τ_xy_) was needed to tighten the adjacent strip layers and to provide enough stiffness to keep the geometric configuration of the STACER to meet its service performance.

**Figure 10:**
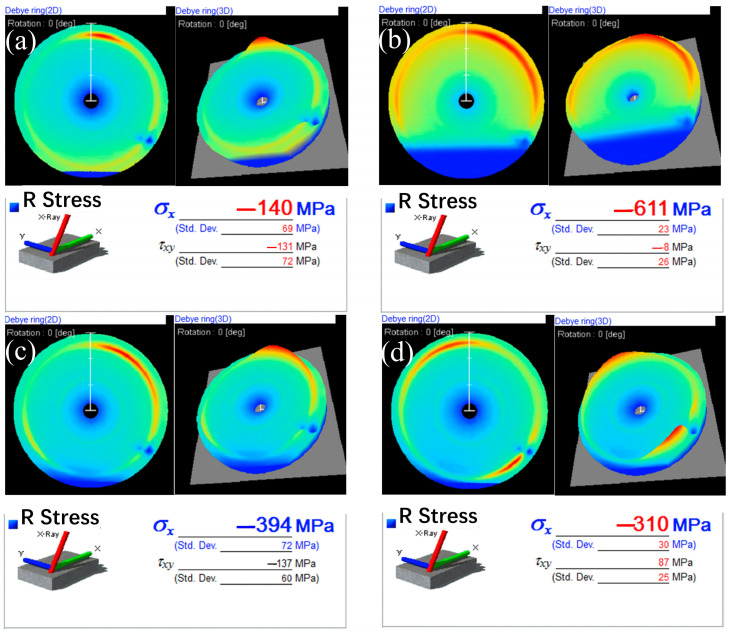
The Debye rings and their residual stresses (σ_x_ and τ_xy_): (**a**) CSPB STACER; (**b**–**d**) winding and stabilization STACER under 480 °C + 4 h, 520 °C + 4 h, and 560 °C + 4 h, respectively.

To further investigate the residual stress distribution, the 2D detector, Pulstec μ-X360n X-ray residual stress analyzer, was adopted to obtain the Debye ring and the residual stress values of the samples. Figure 10 shows the Debye rings and the residual stress (σ_x_ and τ_xy_) of the CSPB STACER’s (Figure 10a) winding and stabilization component under 480 °C + 4 h (Figure 10b), 520 °C + 4 h (Figure 10c), and 560 °C + 4 h (Figure 10d). From Figure 10a, we can see that the residual stress of the CSPB STACER was σ_x_ = −140 MPa, τ_xy_ = −131 MPa; in contrast with the results of the CSPB STACER tested by the sin^2^ Ψ method (Figure 9), they were mostly located around the value −130 MPa, which may indicate that the two residual stress-testing methods (sin^2^ Ψ and cos α) did not make a significant difference in this specific testing process, so we can assume that the two methods have consistency in the residual testing process for STACERs. The residual stress-testing results of the winding and stabilization specimen showed that, with the increase in stabilizing heating temperature, the σ_x_ value of residual stress increased from −611 MPa to −310 MPa, while the τ_xy_ value decreased from −8 MPa to −137 MPa, and then increased to 87 MPa, which indicated that the inner residual stress was released to some extent while keeping the stiffness and shape of the STACER and retaining excellent service performance. The τ_xy_ value of residual stress of the winding and stabilization component under 520 °C + 4 h was in good accordance with the testing result of the CSPB STACER (−128 MPa and −131 MPa), which further confirms that the CSPB STACER and the winding and stabilization STACER under 520 °C + 4 h had consistency in their service performance.

### 3.3. Electron Backscatter Diffraction Analysis

**Figure 11:**
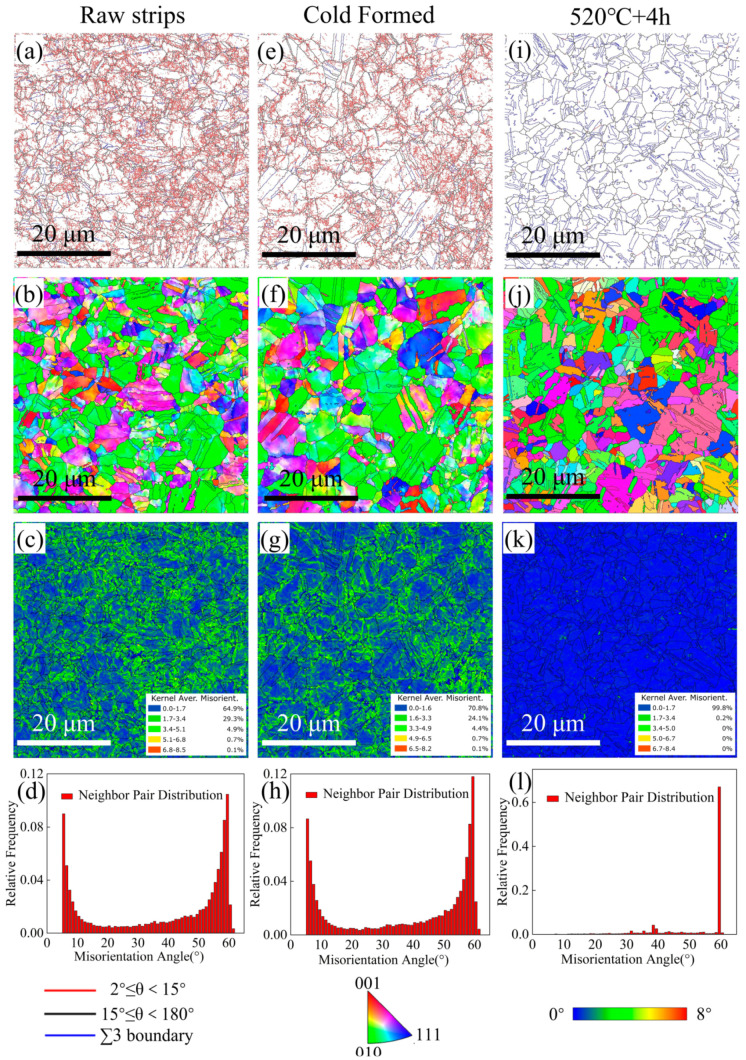
Results of EBSD studies: GB (**a**,**e**,**i**), IPF (**b**,**f**,**j**), KAM (**c**,**g**,**k**), and MAD (**d**,**h**,**l**) maps of raw strips (**a**–**d**), cold-formed STACER (**e**–**h**) and winding and stabilization STACER specimens (**i**–**l**).

The as-received Co40CrNiMo raw strips, cold-formed STACER and the winding and stabilization (520 °C + 4 h) processes were investigated by EBSD technology, and the data of the results were used to construct the corresponding grain boundary (GB), inverse pole figure (IPF), kernel average misorientation (KAM, which shows a difference in the crystal orientations of adjacent measurement points, and generally used to characterize the residual plastic strain), and misorientation angle distribution (MAD) maps as shown in Figure 11. In the GB maps, the low-angle boundaries (LABs) have misorientation angles in the range of 2~15°, the high-angle boundaries (HABs) have misorientation angles greater than 15°, and the ∑3 (<111>60°) boundaries are represented by red, black, and blue lines, respectively (data details in Table 4). The GBs, KAM, and MAD maps exhibit similar trends for the raw strips and cold-formed STACER, which is different from the STACER fabricated through the winding and stabilization process. The LABs, HABs, and ∑3 boundaries account for 66%–34%–20%, 65.4%–34.6%–19.2%, and 1.67%–98.3%–69.1% of the three kinds of specimens, respectively; in particular, the fraction of LABs in the winding and stabilization STACER was close to zero, and the KAM values were much lower than the magnitudes obtained from the specimens of raw strips and the cold–formed STACER. This suggested that the recovery and recrystallization process was initiated in the heat treatment procedure, and reduced the dislocation density by annihilating or rearranging, resulting in the decrease of the LABs and the increase of HABs (mainly the ∑3 (<111>60°) boundaries due to the low SFE in Co40NiCrMo alloy (the twins form easily). It may also result from the formation of deformation-induced boundaries (DIBs) [32] in heavily cold deformed strips, with misorientations evolving from low to high angles with increasing strain and the recovery in heat treatment process, as illustrated in Figure 11. The mean size of the grains calculated by the equivalent circle diameter method from the EBSD data for the three categories of specimen were 2.1, 2.2, and 3.6 μm (Table 4), respectively, indicating that the grains grew by 64% in the heat treatment process.

The as-received Co40NiCrMo strips were provided in the condition of cold rolling under a large reduction of about 70%, resulting in smaller grain sizes with many sub-grains located along the grain boundaries [33]. Since Co40CrNiMo is a face-centered cubic structure alloy with low stacking fault energy (SFE) [34], the raw strips and cold-formed STACERs were prone to recover through the stacking fault interactions and slip dislocations during heat treatment [35]. As shown in Figure 11d,h,i, the “two-peaks” phenomenon disappeared and the misorientation angles around 60° dominated the matrix microstructure—i.e., many more annealing twins appeared.

### 3.4. Transmission Electron Microscopy Analysis

**Figure 12:**
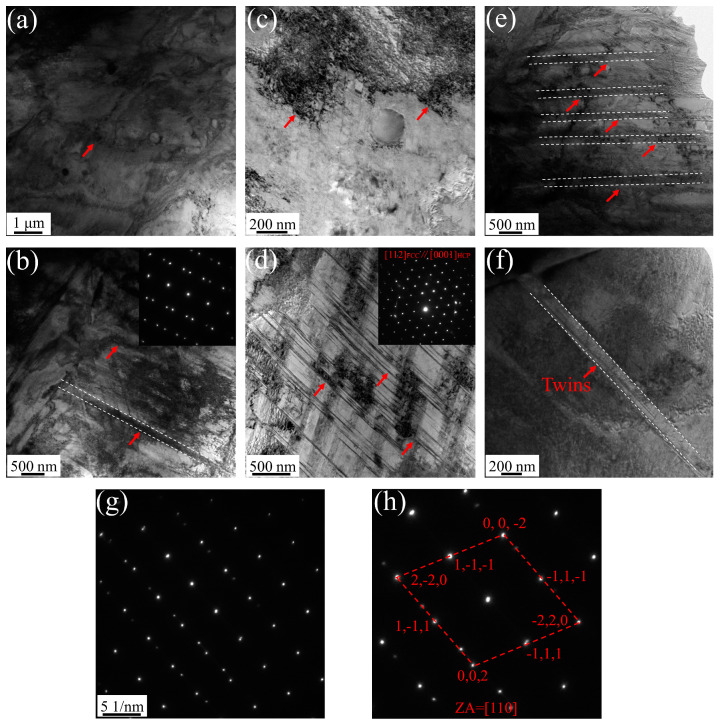
TEM images of Co40NiCrMo strips, Raw strips (**a**,**b**); cold-formed STACER (**c**,**d**); winding and stabilization STACER (520 °C + 4 h) (**e**–**g**) selected-area electron diffraction (SAED) pattern for twins in (**f**); (**h**) indices to (**g**).

**Figure 13:**
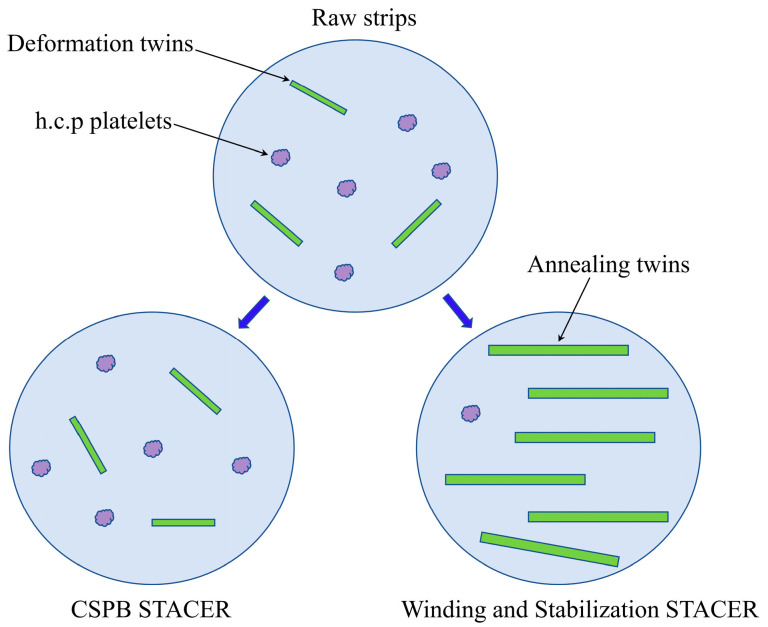
Schematic plot of strengthening mechanisms for raw strips, CSPB STACER, and winding and stabilization STACER.

The specimens from the raw strips, the cold-formed STACER, and the winding and stabilization (520 °C + 4 h) STACER were tested by transmission electron microscopy (TEM); their bright field images and selected area electron diffraction (SAED) patterns are shown in Figure 12. Streaks or “relrods” were observed in the SAED pattern of the samples in raw strips and in the cold-formed STACER; the specimens of these strips were not subjected to the aging process. From Figure 12a–d, the main features were the matrix with many thin platelets; the dark thin platelets that we found were h.c.p. platelets which were identified by their corresponding selected area electron diffraction (SAED) patterns (see Figure 12d); and the diffracted intensity distribution in reciprocal space was extended into streaks perpendicular to their habit planes, so, if the thick dimension of the plate is parallel to the electron beam, the streaks will be recorded on the diffraction pattern, However, some reports [36,37] had indicated that h.c.p ε-platelets were able to form during the aging of the cold-worked Elgiloy alloy, similar to those reported in MP alloys [38,39,40,41], for which aging of the cold-worked samples in the FCC + HCP regions was thought to accelerate the transformation of γ_fcc_ → ε_h.c.p_ platelets [42,43], but the density of the ε phase was not quantified and needed to be studied by further investigation. Aging of the cold-worked strips at a temperature higher than 520 °C and onwards led to softening (induced from the tensile results of samples aging under 560 °C) which was attributed to both the transformation of the deformation-induced h.c.p ε-platelets to the fcc phase (as fcc is stable at high temperature) and the annihilation of the lattice defects produced by the recovery and recrystallization. Due to the as-received strips being severely rolled by the reduction of about 70%, deformation twins also appeared in the specimens (see Figure 12b). This is due to the dislocations, or if the deformation twins were locked by the Suzuki effect while, during the heat treatment process, macroscopic inhomogeneous strain, and t microscopic structural changes, the movable dislocations moved and the shear bands recovered, resulting in the increase of annealing twins and the reduce of h.c.p ε-platelets. From Figure 11i, we can see that the twins accounted for about 68%; further in Figure 12e,f, the twins dominated the majority of the matrix, and the SAED pattern was detected and is indexed in Figure 12g,h, while the thin platelets were fewer than the former two kinds of specimens, and the dislocation decreased significantly (see Figure 11a,e–i). By comparison with Figure 11d,h, the twins in the raw strips and the cold-formed STACER accounted for about 10% and 12%, respectively; we concluded that the strengthening mechanism in the as-received raw strips and cold-formed STACER was the combined action of deformation twins and h.c.p platelets’ networks, while for the strengthening mechanism in the heat–treated STACER (520 °C + 4 h), the twins played a dominant role. The schematic plot of the strengthening mechanisms is shown in Figure 13.

## 4. Conclusions

The as-received Co40NiCrMo alloy strips, cold-formed STACER, and STACER prepared by the winding and stabilization method were subjected to tensile experiments and fracture morphology observation, residual stress measurements, and EBSD and TEM characterization. The main findings of the study are summarized as follows:

(1) The tensile strength/elongation rates were around 1469.04 MPa/20.4%, 1421 MPa/11.5%, and 1562 MPa/5% for the raw strips, CSPB STACER and winding and stabilization STACER, respectively. From SEM fractography of the tensile specimens, the fracture mechanism was determined to be ductile, and the winding and stabilization STACER showed lower ductility combined with the pointing accuracy and driving force service performance; the optimum parameter for the stabilizing heat treatment was determined as 520 °C + 4 h.
(2) The values of residual stress for the raw Co40NiCrMo strips were below 20 MPa; for the cold-formed STACER, the residual stress component τ_xy_ was −128 MPa by the sin^2^ Ψ method (τ_xy_ = −131 MPa by cosα method), which showed consistency with the residual stress component (τ_xy_ = −137 MPa) of the STACER prepared by the winding and stabilization method.
(3) The EBSD and TEM results indicated that the strengthening mechanism of the Co40NiCrMo alloy for the as-received Co40NiCrMo strips and the STACER prepared by the CSPB method was the combined action of deformation twins and h.c.p platelet networks, while for the STACER obtained by the winding and stabilization method, the annealing twins played a dominant role.

## Figures and Tables

**Table 1:** Chemical composition (wt.%) of Co40NiCrMo alloy.

C	Si	Mn	P	S	Cr	Ni	Co	Mo	Fe
0.086	0.24	2.02	<0.01	0.0016	20.14	14.92	40.33	6.90	Bal.

**Table 2:** Tensile strength and elongation of raw strips and CSPB STACER.

Specimen	Tensile Strength (MPa)			Elongation (%)		
	45°	Longitudinal	Transverse	45°	Longitudinal	Transverse
Raw strips	1478.09	1445.83	1483.22	17.92	24.96	18.48
CSPB STACER	1418	1415	1429	12.8	11.6	10.1

**Table 3:** Data of driving force and pointing accuracy for CSPB STACER and winding and stabilization STACER.

Specimen	Driving Force (N)						Pointing Accuracy (°)					
	0 m	1 m	2 m	3 m	4 m	5 m	1	2	3	4	5	6
Cold formed	38.6	29.5	27.5	27.3	24.1	20.5	0.08	0.12	0.1	0.13	0.09	0.11
480 °C + 4 h	28.6	25.5	23.9	23	21.9	19.6	0.55	0.49	0.57	0.62	0.45	0.43
520 °C + 4 h	35.6	28.3	26.8	25.4	22.9	20.1	0.21	0.18	0.22	0.21	0.23	0.19
560 °C + 4 h	27.9	25.8	24.3	23.2	21.1	19.2	0.6	0.46	0.56	0.61	0.52	0.46

**Table 4:** GBs and average grains size of the EBSD results.

Specimen	LABs (%)	HABs (%)	∑3 (<111>60°) (%)	Average Grains Size (μm)
Raw strips	66	34	20	2.1
Cold formed	65.4	34.6	19.2	2.2
520 °C + 4 h	1.67	98.3	69.1	3.6

## Data Availability

Not applicable.

## References

[1] Auslander D., Cermenska J., Dalton G., de la Pena M., Dharan C.K.H., Donokowski W., Duck R., Kim J., Pankow D., Plauche A. (2008). Instrument Boom Mechanisms on the Themis Satellites; Magnetometer, Radial Wire, and Axial Booms. Space Sci. Rev..

[2] Berthelier J.J., Godefroy M., Leblanc F., Malingre M., Menvielle M., Lagoutte D., Brochot J.Y., Colin F., Elie F., Legendre C. (2006). Ice, the Electric Field Experiment on Demeter. Planet Space Sci..

[3] García Marirrodriga C., Pacros A., Strandmoe S., Arcioni M., Arts A., Ashcroft C., Ayache L., Bonnefous Y., Brahimi N., Cipriani F. (2021). Solar Orbiter: Mission and Spacecraft Design. Astron. Astrophys..

[4] Ullrich R., McCauley J., Turin P., McKee K., Donokowski B. (2008). The Stereo Impact Boom. Space Sci Rev..

[5] Bale S.D., Ullrich R., Goetz K., Alster N., Cecconi B., Dekkali M., Lingner N.R., Macher W., Manning R.E., McCauley J. (2008). The Electric Antennas for the Stereo/Waves Experiment. Space Sci. Rev..

[6] Li Z.H. (2017). Research on Stacer Technique for Deployable Boom Device Applied in the Electromagnetic Monitoring Satellite. Ph.D. Thesis.

[7] Li Z.H., Han J.T., Cheng J., Ji S. (2014). Simulation of Residual Stress in Cold Working of Flat Spiral Springs. Adv. Mater. Res..

[8] Wu J., Zhao Z.H., Ren G.X. (2013). Multibody Analysis of the Force in Deploying Booms. J. Guid. Control Dynam..

[9] Li Z.H., Han J.T., Zhang Y.F., Lu R.L., Yang Y. (2023). Research on Forming and Mechanical Properties for One Dimensional Linear Deployable Boom Stacer of Spacecraft. Mater. Today Commun..

[10] Li Z.H., Han J.T., Yu C.Y., Zhang C.F. (2017). Numerical and Experimental Investigation on Forming Stacer Using Compositing Stretch and Press Bending Process. Int. J. Adv. Des. Manuf. Technol..

[11] Yu C.Y., Zhang C.F., Zhang P., Wang S.M. (2016). A Method for Simulating Stacer’s Deployment Deformation. Chin. J. Theor. Appl. Mech..

[12] Li B., Liu Z.Q., Zhang C.F., Yuan D., Yu C.Y., Li X. (2020). Analysis and Verification of Deployed Stiffness of Spiral Tube and Actuator for Controlled Extension and Retraction. J. Astronaut..

[13] Kong N., Li J.Y., Zhang C.F., Zhang J., Li H.B., Wang H.W., Li B., Wang Y. (2020). A Study on the Mechanical Characteristics and Self-Preservation Performance of a Deployment Mechanism with a Large Exhibition Ratio During its Gathering Process. Materials.

[14] Han G.W., Jones I.P., Smallman R.E. (2003). Direct Evidence for Suzuki Segregation and Cottrell Pinning in Mp159 Superalloy Obtained by Feg(S)Tem/Edx. Acta Mater..

[15] Theerthagiri J., Karuppasamy K., Lee S.J., Shwetharani R., Kim H.S., Pasha S., Ashokkumar M., Choi M.Y. (2022). Fundamentals and Comprehensive Insights on Pulsed Laser Synthesis of Advanced Materials for Diverse Photo- And Electrocatalytic Applications. Light: Sci. Appl..

[16] Lee S.J., Theerthagiri J., Nithyadharseni P., Arunachalam P., Balaji D., Madan Kumar A., Madhavan J., Mittal V., Choi M.Y. (2021). Heteroatom-Doped Graphene-Based Materials for Sustainable Energy Applications: A Review. Renew. Sustain. Energy Rev..

[17] Delbergue D., Texier D., Lévesque M., Bocher P. (2019). Diffracting-Grain Identification from Electron Backscatter Diffraction Maps During Residual Stress Measurements: A Comparison Between the Sin 2 Ψ and Cosα Methods. J. Appl. Crystallogr..

[18] Prasad M.J.N.V., Reiterer M.W., Kumar K.S. (2015). Microstructure and Mechanical Behavior of Annealed Mp35N Alloy Wire. Mater. Sci. Eng. A.

[19] Lam A.C.L., Shi Z.S., Lin J.G., Huang X. (2015). Influences of Residual Stresses and Initial Distortion on Springback Prediction of 7B04-T651 Aluminium Plates in Creep-Age Forming. Int. J. Mech. Sci..

[20] Gupta A. (2020). Determination of Residual Stresses for Helical Compression Spring through Debye-Scherrer Ring Method. Mater. Today Proc..

[21] Strodick S., Vogel F., Tilger M., Denstorf M., Kipp M., Baak N., Kukui D., Biermann D., Barrientos M.M., Walther F. (2022). Innovative X-ray Diffraction and Micromagnetic Approaches for Reliable Residual Stress Assessment in Deep Rolled and Microfinished Aisi 4140 Components. J. Mater. Res. Technol..

[22] Shaji E.M., Kalidindi S.R., Doherty R.D. (1999). Influence of Cold-Work and Aging Heat Treatment on Strength and Ductility of Mp35N. Mater. Sci. Eng. A.

[23] Cai Y.Q., Tan Y.B., Wang L.X., Shi W., Ji X.M., Xiang S. (2022). Multiple Strengthening Mechanisms Induced by Nanotwins and Stacking Faults in Conicr-Superalloy Mp159. Mater. Sci. Eng. A.

[24] Fan M., Lyu P., Su Y., Du K., Zhang Q., Zhang Z., Dai S., Hong T. (2021). The Mars Orbiter Subsurface Investigation Radar (Mosir) On China’S Tianwen-1 Mission. Space Sci. Rev..

[25] Otomo T., Matsumoto H., Nomura N., Chiba A. (2009). Influence of Cold-Working and Subsequent Heat-Treatment on Young’s Modulus and Strength of Co-Ni-Cr-Mo Alloy. J. Jpn. Inst. Met..

[26] Zhao Z.Z., Tong T.T., Liang J.H., Yin H.X., Zhao A.M., Tang D. (2014). Microstructure, Mechanical Properties and Fracture Behavior of Ultra-High Strength Dual-Phase Steel. Mater. Sci. Eng, A.

[27] Saeidi N., Ashrafizadeh F., Niroumand B. (2014). Development of a New Ultrafine Grained Dual Phase Steel and Examination of the Effect of Grain Size on Tensile Deformation Behavior. Mater. Sci. Eng. A.

[28] Wang W.R., Li M., Zhao Y.Z., Wei X.C. (2014). Study on Stretch Bendability and Shear Fracture of 800 Mpa Dual Phase Steel Sheet. Mater. Des..

[29] Calcagnotto M., Adachi Y., Ponge D., Raabe D. (2011). Deformation and Fracture Mechanisms in Fine- And Ultrafine-Grained Ferrite/Martensite Dual-Phase Steels and the Effect of Aging. Acta Mater..

[30] Zhong Z., Gu Y., Yuan Y. (2015). Microstructural Stability and Mechanical Properties of a Newly Developed Ni–Fe-Base Superalloy. Mater. Sci. Eng. A.

[31] Ritchie R.O. (2011). The Conflicts between Strength and Toughness. Nat. Mater..

[32] Zolotorevsky N.Y., Rybin V.V., Matvienko A.N., Ushanova E.A., Philippov S.A. (2019). Misorientation Angle Distribution of Deformation-Induced Boundaries Provided by their Ebsd-Based Separation from Original Grain Boundaries: Case Study of Copper Deformed by Compression. Mater. Charact..

[33] Kajima Y., Takaichi A., Kittikundecha N., Nakamoto T., Kimura T., Nomura N., Kawasaki A., Hanawa T., Takahashi H., Wakabayashi N. (2018). Effect of Heat-Treatment Temperature on Microstructures and Mechanical Properties of Co–Cr–Mo Alloys Fabricated by Selective Laser Melting. Mater. Sci. Eng. A.

[34] ISHMAKU A., HAN K. (2004). Deformation Induced Nanostructure and Texture in Mp35N Alloys. J. Mater. Sci..

[35] Rajan K., Vander Sande J.B. (1982). Room Temperature Strengthening Mechanisms in a Co-Cr-Mo-C Alloy. J. Mater. Sci..

[36] Assefpour-Dezfuly M., Bonfield W. (1984). Strengthening Mechanisms in Elgiloy. J. Mater. Sci..

[37] Qureshi I.N., Rani S., Yasmin F., Farooque M. (2010). Tem Study for Strengthening Mechanisms in Elgiloy. Key Eng. Mater..

[38] Ueki K., Ueda K., Narushima T. (2016). Microstructure and Mechanical Properties of Heat-Treated Co-20Cr-15W-10Ni Alloy for Biomedical Application. Metall. Mater. Trans. A.

[39] Ishmaku A., Han K. (2001). Characterization of Cold-Rolled and Aged Mp35N Alloys. Mater. Charact..

[40] Bajpai S., MacDonald B.E., Rupert T.J., Hahn H., Lavernia E.J., Apelian D. (2022). Recent Progress in the Cocrni Alloy System. Materialia.

[41] Gu J., Guo L., Gan B., Bi Z.N., Song M. (2021). Microstructure and Mechanical Properties of an Mp159 Alloy Processed by Torsional Deformation and Subsequent Annealing. Mater. Sci. Eng. A.

[42] Sorensen D., Li B.Q., Gerberich W.W., Mkhoyan K.A. (2014). Investigation of Secondary Hardening in Co–35Ni–20Cr–10Mo Alloy Using Analytical Scanning Transmission Electron Microscopy. Acta Mater..

[43] Achmad T.L., Fu W., Chen H., Zhang C., Yang Z. (2018). Effect of Solute Segregation on the Intrinsic Stacking Fault Energy of Co-Based Binary Alloys: A First-Principles Study. J. Alloys Compd..

